# Pathways to oral health–related quality of life in preschool children: a structural model from SB Brasil 2023

**DOI:** 10.1590/1807-3107bor-2026.vol40.045

**Published:** 2026-07-27

**Authors:** Laissa Viegas Cardoso de BARROS, Jéssica Madeira BITTENCOURT, Raquel Conceição FERREIRA, Saul Martins PAIVA, Cristiane Baccin BENDO

**Affiliations:** (a) Universidade Federal de Minas Gerais – UFMG, School of Dentistry, Department of Pediatric Dentistry, Belo Horizonte, MG, Brazil.; (b) Universidade Federal de Minas Gerais – UFMG, School of Dentistry, Department of Community and Preventive Dentistry, Belo Horizonte, MG, Brazil.

**Keywords:** Quality of Life, Dental Caries, Open Bite, Latent Class Analysis

## Abstract

The aim was to test a structural equation model examining the direct and indirect pathways between early childhood caries (ECC) severity and oral health-related quality of life (OHRQoL) in Brazilian preschool children, incorporating dental pain, treatment urgency, dental visits, anterior open bite (AOB), and maternal schooling into the model. A population-based study was conducted using data on five-year-old children from the 2023 Brazilian National Oral Health Survey (SB Brasil). The Brazilian version of the SOHO-5 was used to measure the impact of oral health conditions on OHRQoL. Calibrated dentists assessed ECC (dmft and pufa indices), AOB (Foster & Hamilton), and urgent oral health care needs. Parents or caregivers answered a questionnaire addressing dental pain, dental visits, and mother’s schooling. Structural equation modeling was performed. Children with greater ECC severity (β = 0.674; p < 0.001) and dental pain (β = 0.241; p < 0.019) were more likely to require urgent care. Greater ECC severity was also directly associated with worse OHRQoL by both by the self-report (β = 0.261; p < 0.001) and proxy-report (β = 0.303; p < 0.001), and indirectly mediated by dental pain in both the self-report (β = 0.426; p < 0.001) and proxy report (β=0.401; p<0.001). Dental pain was associated with worse OHRQoL according to both self-report (β = 0.633; p < 0.001) and proxy-report (β = 0.597; p < 0.001). The structural model demonstrated that ECC severity negatively impacts OHRQoL through direct effects and indirect pathways mediated by dental pain. ECC and dental pain were also associated with a higher likelihood of urgent treatment.

## Introduction

The use of subjective health measures, such as oral health-related quality of life (OHRQoL), has become increasingly relevant for a comprehensive approach to patient care.^1^ Early childhood caries (ECC) and anterior open bite (AOB) are oral conditions that can affect the OHRQoL of children.^2-4^

The multifactorial etiology of ECC is associated with social determinants, which influence health behaviors such as high sugar intake and inadequate oral hygiene.^5,6^ In Brazil, although the prevalence of ECC at five years of age has decreased according to epidemiological surveys conducted in 2003, 2010, and 2023, the rate remains high^7-10^ leading to increased oral morbidity and a negative impact on OHRQoL.^11^ This is due to consequences such as dental pain, poor sleep quality, diminished academic performance, and compromised social interactions in affected children.^2,4,12^

Like ECC, AOB has significant repercussions on the OHRQoL of preschoolers.^3,13,14^ Its etiology is related to hereditary factors, and harmful oral habits, such as digit sucking or prolonged pacifier use, tongue thrusting, mouth breathing, and atypical swallowing.^15^ The most recent Brazilian epidemiological survey revealed that 29.37% of children had some problem in the anterior region of the oral cavity, with a 9.10% rate of anterior open bite.^8^

The Brazilian Ministry of Health has conducted national oral health surveys since 1996, known as SB Brasil since the 2000s.^16^ These surveys contribute to a representative database on the oral health status of the Brazilian population, capturing regional, socioeconomic, and cultural differences and ensuring robust, relevant data for the development and strengthening of public policies.^16^ The measurement of OHRQoL in five-year-old children was incorporated in 2023,^8^ enabling a comprehensive assessment of the impacts of oral conditions on the well-being of Brazilian preschool children.

Previous studies using structural equation modeling in Brazil were conducted with samples that were not nationally representative and included children from age groups different from the index age evaluated in the present study, limiting the generalizability of the findings to the Brazilian population, which is characterized by wide socioeconomic, cultural, and regional heterogeneity. Furthermore, the lack of nationally representative studies that simultaneously integrate a broad range of determinants that can negatively impact OHRQoL, as reported by both parents and children, constitutes a significant gap, especially in countries with great socioeconomic diversity such as Brazil. Therefore, this study advances the understanding of the direct and indirect determinants associated with OHRQoL and provides evidence to serve as a basis for public policies focused on prevention, promotion, and treatment, with the aim of reducing inequalities, improving the effectiveness of children’s oral health programs, and improving the quality of life (OHRQoL) of Brazilian children.

The aim of this study was to test a structural equation model examining the direct and indirect pathways between ECC severity and OHRQoL in Brazilian preschool children, incorporating dental pain, treatment urgency, dental visits, AOB, and maternal schooling into the model. The hypothesis was that dental pain, caries severity, and AOB negatively impact OHRQoL, according to self and proxy reports, and that maternal education is indirectly associated with OHRQoL through the clinical variables.

## Methods

The study is reported following the guidelines of the Strengthening the Reporting of Observational Studies in Epidemiology (STROBE) statement.^17^

### Study design and population

The study used secondary data from the database generated by the 2023 National Oral Health Survey (SB Brasil 2023), a national, cross-sectional survey representative of the entire country.^16,18^ The Federal University of Minas Gerais conducted SB Brasil 2023 and coordinated the research with the Ministry of Health.^16,18^ The study population consisted of five-year-old preschool children (60 to 71 months of age) and their caregivers.

### Ethical aspects

SB Brasil 2023 received approval from the National Research Ethics Committee (approval certificate number 4.823.054). The caregivers of the participants signed an informed consent statement, and the preschool children signed an assent form.^8,16,18^

### Planning and sample calculation

The methodological procedures used in this study are available for public consultation in digital media.^8,16,18,19^ The national survey initially included 7,198 five-year-old preschool children.^8^ The eligibility criterion for this study was individuals with complete data on the variables of interest.

### Latent variable

OHRQoL was measured using the validated Brazilian version of the Scale of Oral Health Outcomes for Five-Year-Old Children (SOHO-5).^16^ The SOHO-5 includes two questionnaires: the child self-report and the parent proxy report. These questionnaires assess the impact of oral conditions on the child’s daily life, including difficulty eating, drinking, speaking, playing, sleeping, smiling for esthetic reasons, and smiling due to pain.^20,21^ Responses to the child self-report are scored on a three-point scale (not at all = 0, a little = 1, a lot = 2) linked to a face scale, with total scores ranging from 0 to 14 points. Responses on the parent proxy report are scored on a five-point scale (not at all = 0, a little = 1, more or less = 2, quite a lot = 3, very much = 4), with an additional “I don’t know” option if the parent or caregiver cannot answer. The total score ranges from 0 to 28 points. The score is calculated by summing the response codes from each questionnaire. Thus, there is a total score to measure OHRQoL according to the self-report and another total score to measure OHRQoL according to the parent proxy report. Higher scores on each questionnaire indicate a greater negative impact on OHRQoL.^20,21^

### Observable variables

#### Clinical variables

ECC was assessed using two indices:^16^ 1) the dmft index (number of decayed, missing, or filled primary teeth);^22^ 2) the pufa index, which assesses the clinical consequences of untreated caries in primary teeth: pulp involvement, ulceration, fistula, and abscess.^23^ For this study, the severity of dental caries was determined by combining the dmft and pufa indices into a single variable (dmft + pufa): tooth without caries experience (code 0); tooth with caries experience and without pulpal consequences (code 1); and tooth with caries experience and pulpal consequences (code 2). A tooth was considered “excluded” when diagnosis was not possible. The dmft index measures cavitated lesions and caries history, while the pufa index identifies infectious complications such as pulpitis, ulceration, and fistulas. Studies suggest that analyzing these indices together provides a more accurate characterization of caries severity and its potential functional impact.^23^

AOB in the primary dentition was measured according to the criteria proposed in the World Health Organization manual (3^rd^ edition),^24^ incorporating the criteria of Foster and Hamilton (1969).^25^ AOB was dichotomized as “absent” or “present”.

The assessment of urgency considered clinical situations,^16^ dichotomized in this study as “no need for treatment, need for preventive or routine treatment, need for elective treatment” (code 0) and “need for immediate treatment (urgency) due to pain or dental/oral infection, need for referral for more comprehensive assessment or medical/dental treatment (systemic condition)” (code 1).^16^

#### Nonclinical variables

All nonclinical observable variables were collected using questionnaires completed by the children’s parents/caregivers.^16,18^ Dental pain was assessed by the intensity of pain reported in the previous six months on a scale from 0 (no pain) to 10 (very severe pain), and was dichotomized as 0 or ≥ 1. Visits to the dentist were assessed with the question, “When did your child (or adolescent) last visit the dentist?”,^16^ and were dichotomized as “has never been to the dentist” (code 0) or “has been to the dentist” (code 1). Socioeconomic status was determined based on maternal education, categorized as follows: did not attend school; took an adult literacy course; incomplete primary school education; complete primary school education; incomplete high school; complete high school; incomplete higher education; complete higher education.^16^ The data collected were entered into software installed on mobile devices, which generated the database for the SB Brasil project.^16^

## Conceptual model

**Figure 1:**
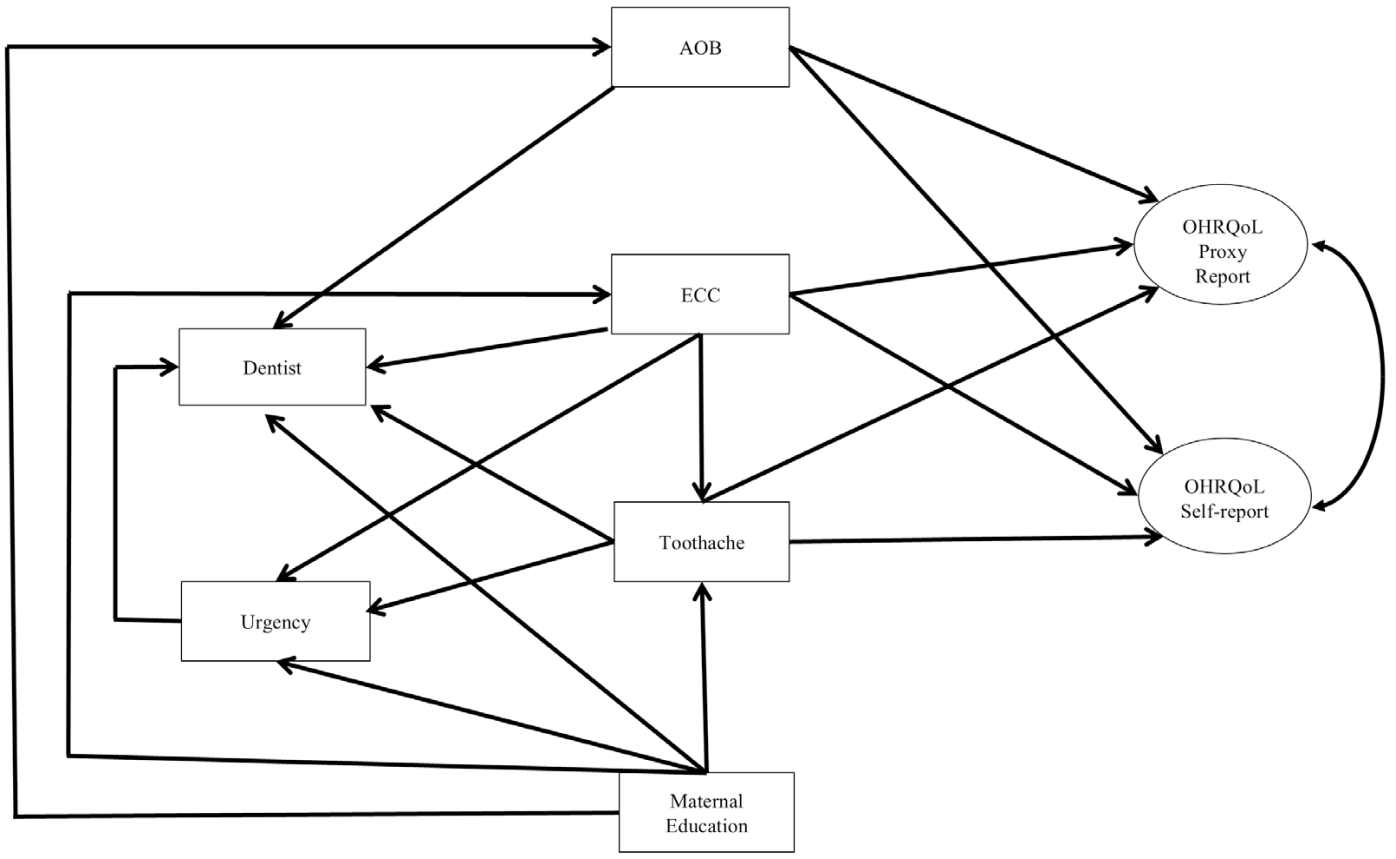
Global theoretical model.

A conceptual model was developed based on previous studies found in the literature,^2,3,4,5,7,12^ and the selection of variables was based on consolidated determinants of oral health in preschoolers (Figure 1). ECC is the most prevalent oral health problem in childhood and is strongly associated with pain, socioeconomic status, and impact on OHRQoL. ^2,4,12^ Dental pain is the main subjective manifestation of caries progression and a direct determinant of negative impacts on OHRQoL. ^2,4^AOB was included because it is an occlusal alteration with possible aesthetic and functional repercussions, already related to psychosocial and well-being impacts.^3^ Although family income is another relevant indicator, SBBRASIL 2023 presented a high proportion of missing data for this variable, compromising its inclusion in the model. Therefore, maternal education was included in the model as it is one of the most consistent determinants of caries risk, health behavior, and use of dental services.^5^ Dental consultation was included as an indicator of access to and use of services, recognized in the literature as a component of childcare, and the need for emergency care reflects complications of untreated conditions and is recognized as a marker of advanced oral conditions in children.^7^

## Data analysis

The data were analyzed descriptively using absolute and relative frequencies as well as measures of central tendency and dispersion, using the IBM SPSS Statistics (IBM SPSS Statistics for Windows, version 26.0, IBM Corp. Released 2019, Armonk,

USA). A measurement model was developed in MPLUS software (version 8.6, Muthén & Muthén, Los Angeles, CA, USA), using confirmatory factor analysis (CFA) to assess the plausibility of the structure of the SOHO-5. Additionally, the residual correlations between items of the SOHO-5 self-report and proxy report questionnaires were examined, considering that values below 0.20 indicate a well-specified factor structure. Subsequently, structural equation modeling (SEM) was performed to create a structural model of ECC, AOB, dental pain, need for urgent care, visit to the dentist, mother’s schooling, and OHRQoL of preschool children. The mediation effect was calculated using the product of the coefficients from the relationship between the independent variable and the mediator, and between the mediator and the dependent variable. Analyses were conducted using the weighted least squares mean and variance-adjusted (WLSMV) estimation method, which is suitable for categorical data.^26-28^ The analysis accounted for sampling weights, as SB Brasil 2023 used a multistage sampling method rather than simple random sampling. Applying the sampling weight was essential to correct potential biases from the complex sampling design, ensuring the validity of inferential estimates for the entire Brazilian population, its regions, states, and major cities. The goodness-of-fit indices used to assess the measurement and structural models were the Comparative Fit Index (CFI), Tucker-Lewis Index (TLI), Standardized Root Mean Residual (SRMR), and Root Mean Square Error of Approximation (RMSEA). CFI and TLI values should be > 0.95 to be considered excellent; RMSEA and SRMR values are considered excellent when < 0.06 and acceptable when between 0.06 and 0.08.^26-28^ The use of SEM enables the creation of complex analytical models capable of identifying direct and indirect relationships between variables. Moreover, SEM recognizes and statistically controls for measurement errors, generating more reliable results.^29,30^

A sensitivity analysis was conducted to assess the robustness of the findings. Two structural models were estimated and compared. Model 1 corresponded to the original analysis using the dataset without imputation of missing values (complete-case analysis). Model 2 was estimated after handling missing data through imputation using expectation maximization. The estimated parameters, structural relationships, and fit indices from Model 2 (imputed dataset) were compared with those from Model 1 (non-imputed dataset) to verify the consistency and stability of the results.

## Results

**Figure 2:**
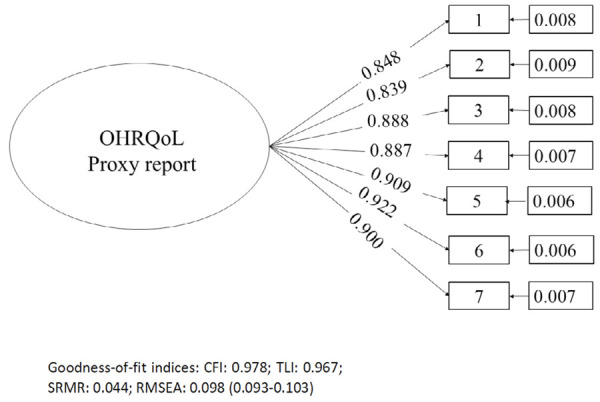
Structure and factor loadings of proxy version of SOHO-5.

**Figure 3:**
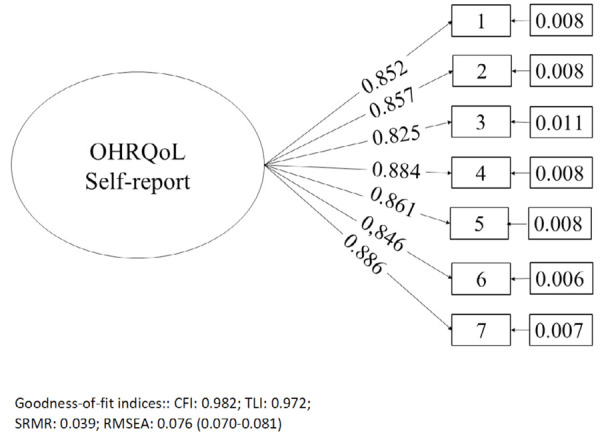
Structure and factor loadings of self-report version of SOHO-5.

A total of 7,198 five-year-old preschool children participated in this study. Descriptive analyses of variables related to oral clinical conditions showed that 13.6% (n = 976) of the children had caries with clinical pulp consequences, 8.4% (n = 545) had AOB, 18.9% (n =1357) had dental pain in the previous months, and 11% (n = 729) required urgent care due to pain (Table 1). The goodness-of-fit indices supported the measurement model of the Brazilian version of the SOHO-5 instrument for both the parent/caregiver proxy report (Figure 2) and the child self-report (Figure 3).

**Table 1:** Distribution of the five-year-old children sample and descriptive analysis of variables before and after data imputation.

Variables	Model 1	Model 2
	without data imputation	with data imputation
	n (%)	n (%)
Regions		
North	1,771 (24.6)	1,771 (24.6)
Northeast	2,638 (36.6)	2,638 (36.6)
Southeast	843 (11.7)	843 (11.7)
South	860 (11.9)	860 (11.9)
Central West	1,086 (15.1)	1,086 (15.1)
Sex		
Male	3,677 (51.1)	3,677 (51,1)
Female	3,521 (48.9)	3,521 (48,9)
ECC*		
Without ECC	3,965 (55.2)	3,974 (55.2)
With ECC/no clinical consequences	2,244 (31.2)	2,247 (31.2)
With ECC and clinical consequences	976 (13.6)	977 (13.6)
AOB*		
Present	545 (8.4)	542 (7.5)
Absent	5,920 (91.6)	6,656 (92.5)
Dental visit*		
Yes	3,994 (58.1)	4,268 (59.3)
No	2,881 (41.9)	2,930 (40.7)
Dental pain*		
Yes	1,357 (18.9)	1,373 (19.1)
No	5,805 (81.1)	5,825 (80.9)
Urgent care due to pain*		
Yes	792 (11)	793 (11)
No	6,393 (89)	6,405 (89)

*Variables with losses in the first model.

**Figure 4:**
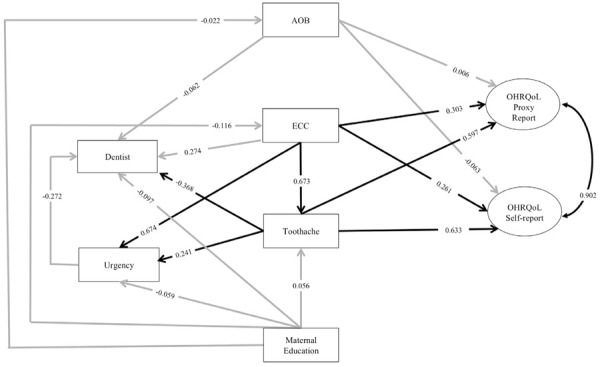
Structural model with standardized coefficients.

The structural model (Model 1) demonstrated an adequate fit, supporting its plausibility. Inspection of standardized residual correlations among items within each questionnaire indicated that residuals were predominantly small (|r| < 0.20), suggesting a well-specified factor structure. Children with greater ECC severity (β = 0.674; p < 0.001) and dental pain (β = 0.241; p < 0.019) were more likely to require urgent care. Greater ECC severity was also directly associated with worse OHRQoL both self-report (β = 0.261; p < 0.001) and proxy-report (β = 0.303; p < 0.001), and indirectly mediated by dental pain in both self-report (β = 0.426; p < 0.001) and proxy report (β = 0.401; p < 0.001). Dental pain was associated with worse OHRQoL according to both self-report (β = 0.633; p < 0.001) and proxy-report (β = 0.597; p < 0.001) (Figure 4; Table 2).

Goodness-of-fit indices: CFI: 0.975; TLI: 0.969; SRMR: 0.073; RMSEA: 0.020 (0.017-0.023). Values in arrows are B values of each association. Direction of association indicated by arrow. Paths in bold: p <0.05. Indirect effect between CPI and QVRSB (self report), mediated by dental pain: B = 0.426; p<0.001; Indirect effect between CPI and QVRSB (proxy report), mediated by dental pain. B = 0.401; p<0.001.

**Table 2:** Standardized coefficients of structural equation modeling (Model 1 without data imputation).

Variables	Model 1 without data imputation	95%CI	Error
SOHO-5 Proxy report			
Dental pain	0.597 (< 0.001)	0.493 to 0.700	0.063
AOB	0.006 (0.942)	-0.130 to 0.142	0.083
ECC	0.303 (< 0.001)	0.190 to 0.417	0.069
SOHO-5 Self-report			
Dental pain	0.633 (< 0.001)	0.552 to 0.715	0.049
AOB	-0.063 (0.449)	-0.200 to 0.074	0.083
ECC	0.261 (< 0.001)	0.169 to 0.354	0.056
Urgent care			
ECC	0.674 (< 0.001)	0.536 to 0.811	0.084
Dental pain	0.241 (0.019)	0.072 to 0.410	0.103
Maternal education	-0.059 (0.470)	-0.193 to 0.075	0.082
Dental pain			
ECC	0.673 (< 0.001)	0.588 to 0.757	0.051
Maternal education	0.056 (0.417)	-0.057 to 0.168	0.069
ECC			
Maternal education	-0.116 (0.081)	-0.226 to -0.007	0.067
Dental visit			
ECC	0.274 (0.104)	-0.004 to 0.551	0.169
Dental pain	-0.368 (0.007)	-0.594 to -0.143	0.137
Urgent care	-0.272 (0.236)	-0.650 to 0.106	0.230
AOB	-0.062 (0.593)	-0.239 to 0.115	0.108
Maternal education	-0.097 (0.118)	-0.198 to 0.005	0.062
AOB			
Maternal education	-0.022 (0.668)	-0.107 to 0.063	0.052
General fit indices			
CFI	0.975	-	-
TLI	0.969	-	-
SRMR	0.073	-	-
RMSEA	0.020 (0.017–0.023)	-	-

AOB: anterior open bite; ECC: early childhood caries; CFI: comparative fit index; RMSEA: root mean square error of approximation; SRMR: standardized root mean square residual; TLI: Tucker-Lewis Index.

In the sensitivity analysis, Model 2 (estimated using the imputed dataset) yielded results consistent with those observed in Model 1 (complete-case analysis). The direction, magnitude, and statistical significance of the main structural paths remained unchanged, and the overall model fit indices were similar for both models. The only additional significant associations identified in Model 2 were between maternal education and dental pain (β = -0.088; p = 0.033), maternal education and ECC (β = -0.218; p < 0.001), and urgency care and visit to dentist (β = 0.351; p = 0.041) (Table 3).

**Table 3:** Standardized coefficients of structural equation modeling (Model 2 with data imputation).

Variables	Model 2 with data imputation	95%CI	Error
SOHO-5 Proxy report			
Dental pain	0.721 (< 0.001)	0.645 to 0.797	0.046
AOB	0.064 (0.568)	-0.120 to 0.249	0.112
ECC	0.128 (0.016)	0.040 to 0.216	0.053
SOHO-5 Self-report			
Dental pain	0.698 (<0.001)	0.627 to 0.770	0.043
AOB	-0.033 (0.685)	-0.168 to 0.102	0.082
ECC	0.108 (0.016)	0.029 to 0.188	0.053
Urgent care			
ECC	0.741 (< 0.001)	0.655 to 0.827	0.052
Dental pain	0.179 (0.005)	0.074 to 0.284	0.064
Maternal education	0.021 (0.629)	-0.051 to 0.093	0.044
Dental pain			
ECC	0.679 (< 0.001)	0.625 to 0.733	0.033
Maternal education	-0.088 (0.033)	-0.156 to -0.020	0.041
ECC			
Maternal education	0.021 (0.629)	-0.278 to -0.158	0.037
Dental visit			
ECC	-0.162 (0.277)	-0.408 to 0.083	0.149
Dental pain	-0.400 (< 0.001)	-0.559 to -0.240	0.097
Urgent care	0.351 (0.041)	0.068 to 0.634	0.172
AOB	-0.248 (0.405)	-0.739 to 0.242	0.298
Maternal education	-0.429 (0.056)	-0.798 to 0.060	0.224
AOB			
Maternal education	-0.493 (0.123)	-1.018 to 0.033	0.320
General fit indices			
CFI	0.975	-	-
TLI	0.969	-	-
SRMR	0.174	-	-
RMSEA	0.019 (0.017–0.021)	-	-

AOB: anterior open bite; ECC: Early Childhood Caries; CFI: comparative fit index; RMSEA: root mean square error of approximation; SRMR: standardized root mean square residual; TLI: Tucker-Lewis Index.

## Discussion

This study analyzed a structural model of ECC, AOB, dental pain, need for urgent care, dental visits, mother’s schooling, and OHRQoL using a representative database of five-year-old preschool children from all regions of Brazil. For the first time, OHRQoL was measured for this age group in a national survey, considering the potential impacts of oral conditions on the daily activities of preschool children. The results reveal that ECC and dental pain have a greater impact on OHRQoL because these oral conditions interfere with essential activities such as eating, playing, and sleeping, significantly affecting children’s routines and general well-being.^2,12,15^ This study proposes a complex structural model that overcomes the limitations of classical analyses by enabling the inclusion of all variables in a single model for simultaneous analysis.^28-30^

The negative impact of ECC on OHRQoL is well established in the literature ^2,4,12,15^ and is often linked to clinical complications resulting from untreated ECC, such as fistulas and abscesses.^2,15^ Thus, the present study analyzed the severity of ECC by considering the presence of clinical consequences of untreated caries as the worst condition. Notably, this is the first representative study of all of Brazil to assess OHRQoL in five-year-old children, enabling the extrapolation of results to the entire population of children in this age group in the country. Moreover, based on evidence demonstrating that preschool children can report OHRQoL in a valid and reliable manner, the use of the SOHO-5 constitutes an advance by enabling both self-reports from children and proxy reports from their parents or caregivers for a more comprehensive understanding of OHRQoL in this population.^20,21^

The present study demonstrated that dental pain mediated the relationship between ECC and poorer OHRQoL, and was also associated with visits to the dentist. A previous study found that difficulty eating had the greatest impact in self-reports, and this difficulty was associated with dental pain.^15^ Pain can compromise daily activities and social interactions, making it a common reason for seeking dental care.^11^ In preschool children, the occurrence and perception of dental pain are associated with several factors, especially the presence of caries, the need for dental treatment, and unfavorable socioeconomic status.^32^

In the imputed data model (Model 2), the identification of associations between maternal education, dental caries, and dental pain suggests that sample losses in the original model may have limited the detection of these effects. Maternal education is a widely recognized social determinant of children’s oral health, influencing health practices, access to dental services, and exposure to risk factors during childhood. Multiple imputation allowed the incorporation of information from individuals with missing data, increasing the statistical power and precision of the model estimates. Thus, Model 2 proved to be more sensitive in capturing socioeconomic inequalities, revealing associations that may have been underestimated in the analysis due to incomplete data.

Among the main strengths of this study is the use of SEM, which overcomes the limitations of classical analyses, enabling the inclusion of all variables in a single model for simultaneous analysis as well as the estimation of the direction of associations and the determination of indirect effects.^28,29,30^ Although the use of SEM allows testing theoretical pathways, the results should be interpreted as associations rather than causal effects.

This study has limitations that should be considered when interpreting the findings. Although the sample is national and representative, the collection of reports may introduce information bias, especially in relation to the SOHO-5, which captures distinct perceptions of caregivers and children and may result in discrepancies and possible underestimations or overestimations of oral impacts. In addition, relevant socioeconomic variables, such as family income, had missing data and were not available with adequate quality to be included in the model, as well as some categories of the variables for dental visits and emergencies. This limited the socioeconomic characterization to maternal education and the dichotomization of the variables dental visits and emergencies. Thus, the model included only one socioeconomic indicator, which may not fully capture the multidimensional nature of social inequalities in oral health. Despite these limitations, the use of a large and nationally representative dataset and a robust analytical approach reinforce the reliability and generalizability of the results.

The results of this study underscore the need for a more comprehensive clinical approach that includes not only diagnosing the main health problems affecting preschool children but also considering the impact on OHRQoL. Healthcare providers should implement health promotion and prevention measures to increase awareness among patients and their parents/caregivers. Given the importance of data from a Brazilian national survey, it is important to propose collective measures such as expanding access to fluoridated water, conducting nutritional education campaigns in schools, and regulating the advertising of ultra-processed foods and those high in sugar content targeted at children. Promoting healthy school environments by offering balanced meals and restricting products rich in added sugars is also an important public health action. Therefore, integrating clinical, educational, and collective actions is essential to promote children’s oral health equitably, prevent complications, and contribute to the overall development and improvement of OHRQoL in preschool children.

## Conclusion

The structural model demonstrated that the severity of ECC negatively impacts OHRQoL through both direct effects and indirect pathways mediated by dental pain. ECC and dental pain were also associated with a higher likelihood of urgent treatment.

## Data Availability

The authors declare that all data generated or analyzed during this study are included in this published article.
